# MicroRNA-153 promotes Wnt/β-catenin activation in hepatocellular carcinoma through suppression of WWOX

**DOI:** 10.18632/oncotarget.2927

**Published:** 2015-02-02

**Authors:** Hong-Wei Hua, Feng Jiang, Qian Huang, Zhijun Liao, Gang Ding

**Affiliations:** ^1^ Xinhua Hospital Affiliated to Shanghai Jiao Tong University School of Medicine, Shanghai 200092, China; ^2^ Department of Oncology, Xinhua Hospital Affiliated to Shanghai Jiao Tong University School of Medicine, Chongming Branch, Shanghai 202150, China

**Keywords:** Hepatocellular carcinoma, β-catenin, MicroRNA, MicroRNA-153, WWOX

## Abstract

Persistent activation of Wnt/β-catenin signaling plays crucial roles in the development of human cancers, including hepatocellular carcinoma (HCC). Here, we performed a MicroRNA-based genetic screen, which revealed a novel diversion in β-catenin signaling triggered by MicroRNA-153 (miR-153). Overexpression of miR-153 was able to promote β-catenin transcriptional activity, leading to cell-cycle progression, proliferation and colony formation of HCC cells. Additionally, systemic administration of miR-153 antigomir suppressed hepatocellular carcinogenesis in a murine liver cancer model. At the molecular level, we found that miR-153 inhibited protein level of WWOX, a tumor suppressor and inhibitor of β-catenin signaling, through targeting its 3′-untranslated region. Therefore, our study highlights the importance of MicroRNA-153/WWOX/β-catenin regulatory axis in the HCC tumorigenesis.

## INTRODUCTION

Hepatocellular carcinoma (HCC), the major type of liver cancers, has become one of the most common causes of cancer mortality worldwide [1, 2]. Previous studies have found that multiple oncogenes or tumor suppressors, such as NF-κB, FoxO1 and PTEN, are involved in the HCC initiation and (or) progression [3–5]. However, the molecular mechanisms underlying the pathogenesis of HCC remain to be defined.

The inappropriate and persistent activation of Wnt/β-catenin signaling pathway plays a key role in the proliferation and cell-cycle progression of HCC [6]. The accumulation of nuclear β-catenin and the up-regulated transcriptional activity of the β-catenin were observed in HCC tissues and cell lines [7]. Moreover, a recent study demonstrated that Wnt/β-catenin pathway activation is sufficient for malignant transformation of liver progenitor cells [8], suggesting that small-molecule inhibitor or antagonist of Wnt signaling may have strong implications for HCC treatment [9].

It has been shown that transcriptional activity or protein stability of β-catenin is tightly regulated by several tumor suppressors, including adenomatous polyposis coli (APC), WTX, PTEN and Menin [10–13]. Interestingly, recent studies suggest that β-catenin-mediated transcription could be regulated by MicroRNAs [14], a class of single-stranded non-coding RNA molecules [15], which shed light on the precise regulation of Wnt/β-catenin signaling in tumorigenesis. In the present study, we performed a MicroRNA-based genetic screen, which revealed a novel diversion in β-catenin signaling triggered by miR-153 in HCC development. Our results further indicate that miR-153-medicated activation of β-catenin may play an important role in the HCC progression.

## RESULTS

### MicroRNA-153 promotes β-catenin signaling in HCC cells

**Figure 1:**
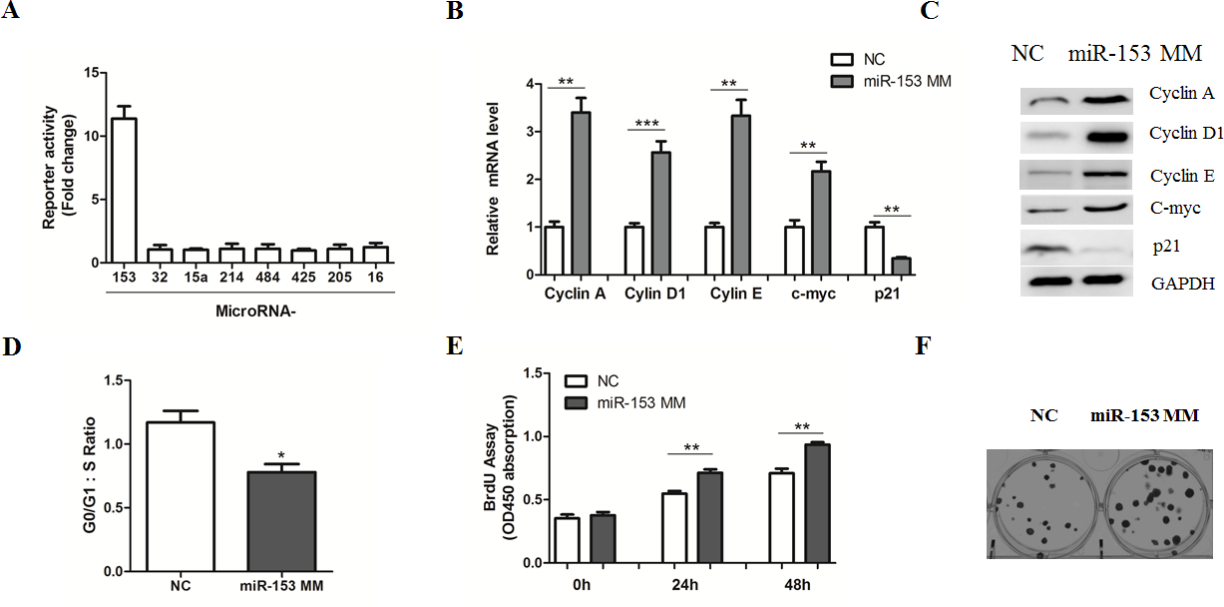
MicroRNA-153 mimics promotes β-catenin signaling in HCC cells **(A)** HepG2 cells transiently transfected with the TopFlash–FopFlash and the indicated MicroRNAs for 36 hr. Luciferase values were measured using the Dual-Luciferase Reporter Assay System and renilla luciferase gene was used to normalize the transfection efficiency. **(B–C)** Relative mRNA (B) and protein (C) levels of Cyclin A, Cyclin D1, Cyclin E, c-myc and p21 were determined in HepG2 cells transfected with miR-153 mimics (MM) or negative controls (NC) for 24 or 48 hr, respectively. **(D)** Cell-cycle analysis of HepG2 cells transfected with NC or miR-153 mimics. Cells were stained with propidium iodide and analyzed by flow cytometry. **(E–F)** Cell proliferation (E) and colony formation (F) assays by transfection of NC or miR-153 mimics.

**Figure 2:**
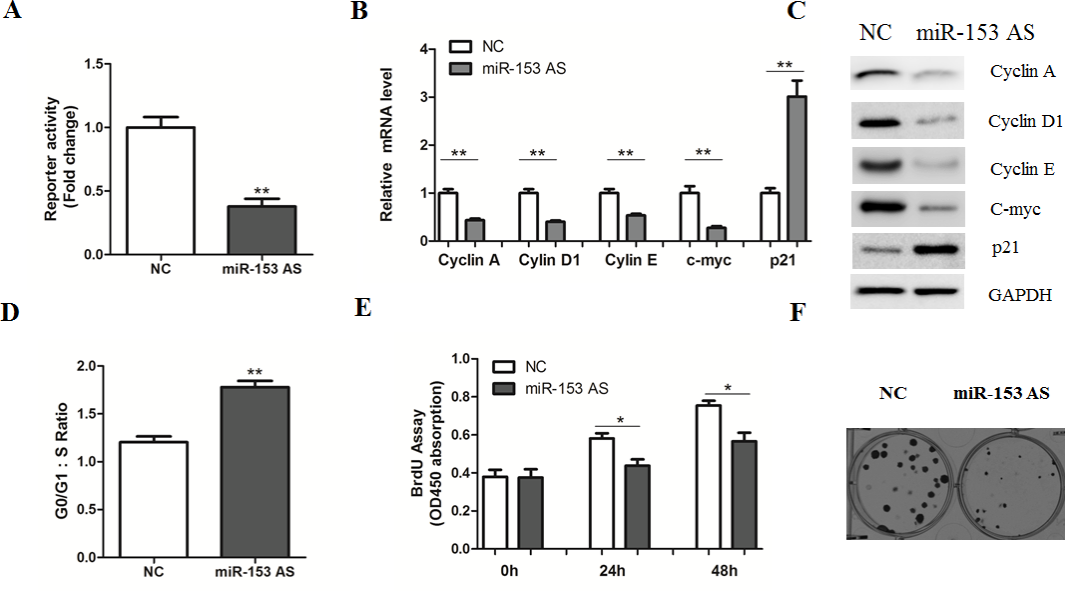
MicroRNA-153 antisense inhibits β-catenin signaling **(A)** HepG2 cells transiently transfected with the TopFlash–FopFlash and miR-153 antisense (AS) or NC for 36 hr. **(B–C)** Relative mRNA (B) and protein (C) levels of Cyclin A, Cyclin D1, Cyclin E, c-myc and p21 were determined in HepG2 cells transfected with miR-153 AS or negative controls (NC) for 24 or 48 hr, respectively. **(D)** Cell-cycle analysis of HepG2 cells transfected with NC or miR-153 AS. **(E–F)** Cell proliferation (E) and colony formation (F) assays by transfection of NC or miR-153 AS.

Given the critical roles of β-catenin signaling in the stimulation of HCC cell proliferation [5–7], we performed a microRNA-based genetic screen to examine whether certain miRNAs could regulate β-catenin activity using the TopFlash reporter system in HepG2 cells. As a result, transfection of miR-200a and miR-135a mimics could inhibit the reporter activity (Data not shown), which is consistent with previous reports [16, 17]. Notably, we found that overexpression of miR-153 mimics resulted in a substantial increase of reporter activity (Figure 1A), which was accompanied by the up-regulation of Cyclin A, Cyclin D1, Cyclin E and C-myc, as well as the reduction of p21, leading to an increased cell cycle progression, cell proliferation and colony formation (Figure 1B–1F). In agreement, inhibition of miR-153 by its antisense oligos led to a suppression of β-catenin activity (Figure 2A). The expression levels of Cyclin A, Cyclin D1, Cyclin E, C-myc and p21 were also affected by miR-153 antisense (Figure 2B–2C), accompanied with a reduction of cell growth and colony formation (Figure 2D–2F). Therefore, our observations suggest a novel mechanism for MicroRNA-induced cell-cycle progression triggered through the promotion of β-catenin-mediated transcription.

### MicroRNA-153 regulates β-catenin activation through suppression of WWOX

**Figure 3:**
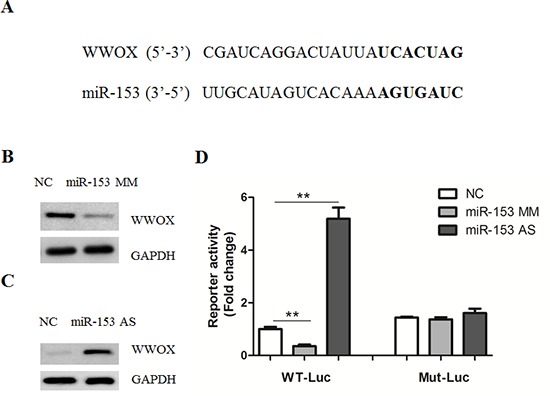
MicroRNA-153 regulates WWOX expression **(A)** Computer prediction of miR-163 binding sites in the 3′-UTRs of human WWOX genes. Potential binding sites were highlighted in bold. **(B–C)** Western blot analysis of WWOX expression in HepG2 cells transfected with miR-153 mimics (B), antisense (C) or negative control (NC). **(D)** Luciferase reporter assays in HepG2 cells. Cells were transfected with 100 ng of wild-type 3′-UTR-reporter or mutant constructs together with miR-153 mimics, antisenseor negative control (NC).

Next, we examined the mechanisms underlying the inhibitory effect of miR-153 on β-catenin-dependent gene transcription. To this end, bioinformatics software (TargetScan) was employed to identify potential target genes for miR-153. Among which, we found that WWOX, harbored a potential miR-153 binding site in its 3′-untranslated region (3′-UTR) (Figure 3A). Transfection of miR-153 mimics resulted in a reduced WWOX protein expression (Figure 3B and Supplementary Figure 1A). In agreement, a dramatic up-regulation of WWOX was observed in cells with miR-153 inhibition (Figure 3C and Supplementary Figure 1B). It has been shown that enforced WWOX expression inhibited, and suppression of endogenous WWOX expression stimulated the transcriptional activity of β-catenin, by preventing the nuclear import of the Dishevelled-2 (Dvl-2) protein [18]. In agreement, we found that overexpression of miR-153 mimics promoted, while its antisense inhibited the nuclear localization of Dvl-2 protein (Supplementary Figure 2A–2B).

To understand how miR-153 regulates WWOX expression, the luciferase reporter plasmids containing the 3′-UTR of WWOX was co-transfected with miR-153 mimics or antisense. As shown in Figure 3D, miR-300 mimics or antisense led to a reduction or increase of luciferase activity. Furthermore, mutagenesis of the seed sequence abolished the effects of miR-153 mimics or antisense on WWOX activity (Figure 3D). To further verify the functional connection between miR-153 and WWOX, HepG2 cells were transfected with adenovirus containing WWOX or GFP as a negative control (Supplementary Figure 3A). As a result, WWOX overexpression reversed the oncogenic roles of miR-153 (Supplementary Figure 3B–3C), underlining the specific importance of the WWOX for miR-153 action in the cell proliferation.

### MicroRNA-153 promotes HCC growth *in vivo*

**Figure 4:**
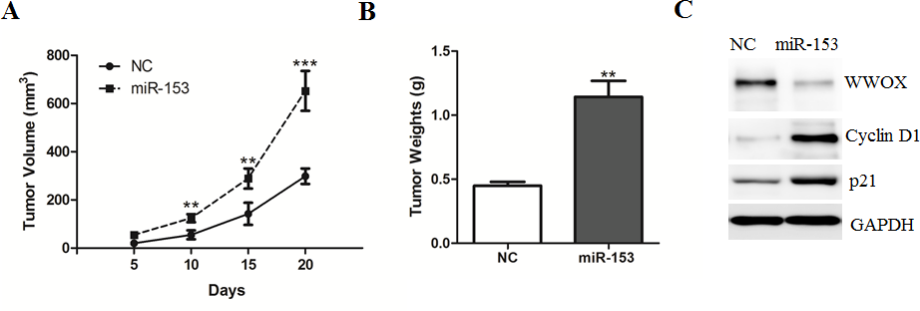
MicroRNA-153 promotes HCC growth in the nude mice **(A–B)** HepG2 cells stably transfected with miR-153 or negative control (NC) were injected into nude mice (*n* = 6–8 for each group) and followed up for tumorigenesis. Growth curve of tumor volumes (A) and tumor weights (B) were taken 20 days after injection. **(C)** Representative protein levels of WWOX, Cyclin D1 and p21 were determined in the two groups of tumors.

To further demonstrate its function, we tested if forced expression of miR-153 promotes the ability of HepG2 cells to form xenograft tumors in nude mice. We injected approximately 5 × 10^6^ stable HepG2 cells subcutaneously into two bilateral sites on the lower back of 5 weeks old BALB/c nude mice. The tumors were measured weekly for 4 consecutive weeks, and each tumor was individually weighed after the mice were euthanized. As a result, the tumor volume and weight were markedly increased in miR-153-overexpressed tumors compared to control tumors (Figure 4A–4B). In addition, we observed a reduced protein levels of WWOX and p21, an increased expression of Cyclin D1 by miR-153 overexpression (Figure 4C), suggesting that miR-153 could also promote tumor growth *in vivo*.

**Figure 5:**
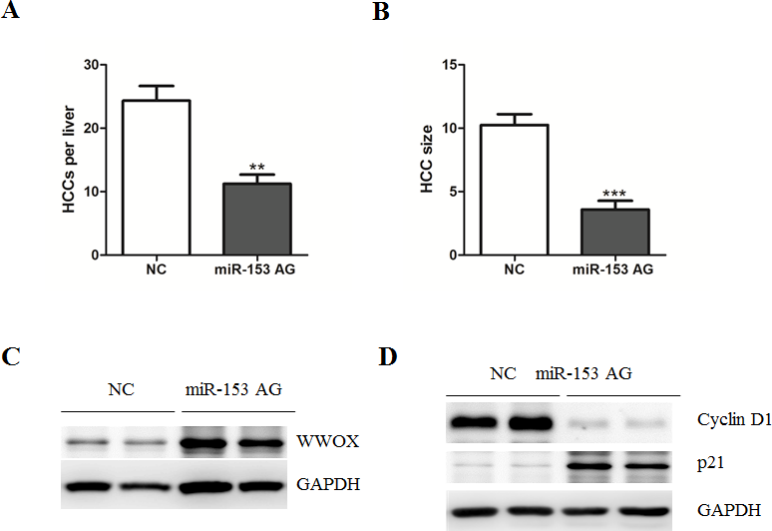
Systematic administration of MicroRNA-153 suppresses HCC development in C57BL/6 mice **(A–B)** Number of HCC tumors/liver (A) and tumor size (mm^3^) (B) in NC-, and miR-153 antigomir-treated mice. **(C–D)** Representative protein levels of WWOX, Cyclin D1 and p21 were determined in the two groups of tumors.

Next, we asked whether miR-153 inhibition has therapeutic and preventive effects for HCC using a murine liver cancer model. It is well-known that diethylnitrosamine (DEN)-treated mice developed tumors spontaneously [19]. As shown in the Supplementary Figure 4, expression levels of miR-153 gradually increased in C57BL/6 mice treated with DEN, compared with vehicle controls. Based on this finding, we treated mice with systemically administration of miR-NC or miR-153 antagomir at week 28. On week 35, the mice were sacrificed and the tumors were analyzed. As expected, inhibition of miR-153 dramatically suppressed HCC growth and size (Figure 5A–5B). Importantly, we found that systemic delivery of miR-153 antagomir up-regulated WWOX protein levels and inhibited expression of Wnt signaling target genes, including c-myc and Cyclin D1 (Figure 5C–5D).

### MicroRNA-153 correlates with poor survival of HCC patients

**Figure 6:**
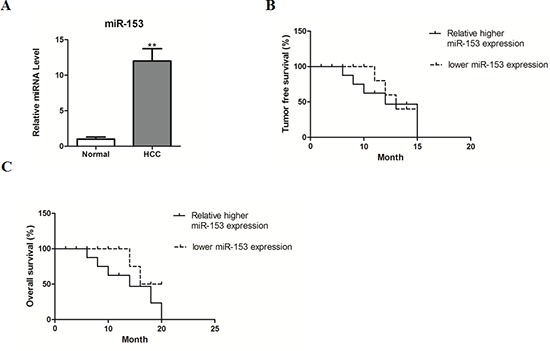
MicroRNA-153 correlates with poor survival of HCC patients **(A)** miR-153 expression was determined by real-time PCR in human HCC tissues and adjacent normal tissues. **(B–C)** Kaplan-Meier survival curves of tumor-free survival (B) and overall survival C) according to the ratio of miR-153 level in each HCC sample compared with its matched non-cancerous control, the median value of this ratio in each cohort was chosen as the cutoff point.

To further investigate whether the deregulated abundant miR-153 correlates with the survival of HCC patients, expression levels of miR-153 were determined in HCC and matched non-cancerous tissues. As expected, miR-153 was significantly up-regulated in HCC tissues, compared with adjacent normal tissues (Figure 6A). Kaplan-Meier analysis further revealed that low miR-153 level in HCC tissues significantly correlated with the markedly reduced tumor-free survival and overall survival of HCC patients (Figure 6B–6C).

## DISCUSSION

Previous studies have identified Wnt/β-catenin signaling as a direct and functional target of several miRNAs, such as miR-200a, miR-135a, miR-30–5p and miR-612 [16, 17, 20, 21]. In the present study, our data showed that miR-153 could be a novel and important regulator of β-catenin signaling in HCC. These are supported by multiple lines of evidence. First, overexpression of miR-153 promoted, while its antisense inhibited the transcriptional activity of β-catenin and expression of its down-stream target genes. Second, miR-153 could regulate cell proliferation and tumor growth *in vitro* and *in vivo*. Third, miR-153 was up-regulated in HCC tissues and correlated with poor survival of patients. Therefore, our data present a novel mechanism for the persistent Wnt signaling activation in tumorigenesis.

At the molecular level, our luciferase reporter and Western blot analysis found that miR-153 could suppress WWOX expression through targeting its 3′-UTR region. It has been shown that dys-regulation of WWOX could contribute to genomic instability, cancer progression and therapy resistance [22, 23]. Indeed, WWOX heterozygous knockout mice exhibited a higher incidence of spontaneous or chemically induced tumors [24, 25]. Besides, WWOX expression was down-regulated in several types of human cancer tissues cell lines, including pancreatic adenocarcinoma, renal cell carcinoma, endocrine tumors and HCC [26, 28]. At the molecular level, WWOX was involved in cell-cycle progression and apoptotic process by interacting with the proteins that contain PPxY motifs, such as ErbB4, p73, and C-Jun [29–31]. Besides, Bouteille N et al. found that enforced WWOX expression inhibited, and inhibition of endogenous WWOX expression stimulated the transcriptional activity of the Wnt/β-catenin pathway, at least in part, by preventing the nuclear import of the Dishevelled protein [18]. Therefore, elucidating the signaling network to shed light upon the role of WWOX may help to understand the HCC biology and identify potential therapeutic targets.

It has been reported that miR-153 was down-regulated and correlated significantly with advanced clinical stage in ovarian cancers [32], suggesting that miR-153 might be of potential importance as diagnostic biomarkers. Besides, up-regulation of miR-153 promotes cell proliferation via suppression of the PTEN tumor suppressor gene in human prostate cancer [33]. Moreover, miR-153 supports colorectal cancer progression via pleiotropic effects that enhance invasion and chemotherapeutic resistance [34]. On the other hand, overexpression of miR-153 significantly inhibited the proliferation and migration, and promoted apoptosis of lung cancer cells, through suppression of AKT expression [35]. Therefore, miR-153 could either act as an onco-miRNA or a tumor suppressor in human cancers, which might be dependent on cellular context. However, the molecular determinants of miR-153 up-regulation in HCC tissues remain to be determined.

Taken together, in the present study, our results highlight the roles of miR-153 in the regulation of HCC. Further studies are still needed to investigate its biological function in tumorigenesis by modern technology, such as miR-153 transgenic or liver-specific knockout mice.

## MATERIALS AND METHODS

### Human tissue samples

42 parried of HCC tissues and adjacent non-tumor normal tissues were collected from routine therapeutic surgery at our department. All samples were obtained with informed consent and approved by the hospital institutional review board.

### Cell culture

HCC cell lines (HepG2 and HuH7 cells) were obtained from The Cell Bank of Type Culture Collection of Chinese Academy of Sciences (CAS, Shanghai). Cells were grown in Dulbecco's modified Eagle's medium (DMEM, Gibco, Shanghai) supplemented with 10% fetal bovine serum (Gibco) and maintained at 37°C in a humidified atmosphere with 5% CO2.

### Mouse experiments

Male BALB/c nude mice aged 4 weeks were purchased from Shanghai Laboratory Animal Company (SLAC, Shanghai). 5.0 × 10^6^ HepG2 cells stably expressing miR-153 or negative control (miR-NC) were injected subcutaneously to the skin under the front legs of the mouse. The mice were observed over 20 days for tumor formation. After the mice were sacrificed, the tumors were recovered and the wet weights of each tumor were determined.

To induce hepatocellular carcinogenesis in C57BL/6 mice, mice were injected intraperitoneally with 25 mg/kg of diethylnitrosamine (DEN) (Sigma-aldrich) at 21 days of age. Mice were systemically administrated with miR-NC or miR-153 antagomir at week 28. On week 35, the mice were sacrificed and the tumors were analyzed.

### MicroRNA real-time PCR analysis

Total RNA from tissues and cells was extracted using the miRNA Isolation Kit (Ambion, USA) according to the manufacturer's instructions. Expression of mature miRNAs was assayed using Taqman MicroRNA Assay (Applied Biosystems, USA). Quantitative real-time PCR was performed by using an Applied Biosystems 7300 Real-time PCR System and a TaqMan Universal PCR Master Mix. Expression of the miRNAs was normalized to that of the U6 snRNA.

### Western blot

Cells were harvested and lysed with ice-cold lysis buffer (50 mM Tris-HCl, pH 7.4, 100 mM 2-Mercaptoethanol, 2% w/v SDS, 10% glycerol). After centrifugation at 10000 × g for 10 min at 4°C, proteins in the supernatants were quantified and separated by 10% SDS PAGE. Western blot assay was performed using anti-WWOX, β-Catenin, Cyclin A, Cyclin D1, Cyclin E, c-myc and p21 antibodies (Abcam, USA). Protein levels were normalized to total GAPDH, using a rabbit anti-GAPDH antibody (Santa Cruz, USA).

### Transfection and luciferase reporter assay

miR-153 mimics and antisense were purchased from Ambion Company (Invitrogen, USA). The 3′-untranslated region of human WWOX gene was cloned into pMir-Report plasmid (Ambion), yielding pMir-Report-WWOX. Mutations were introduced in potential miR-153 binding sites using the QuikChange site-directed mutagenesis Kit (Stratagene, USA). Transfection was performed with Lipofectamine 2000 (Invitrogen) according to the manufacturer's recommendations. The pRL-TK vector (Promega, USA) carrying the Renilla luciferase gene was used to normalize the transfection efficiency. Luciferase values were measured using the Dual-Luciferase Reporter Assay System (Promega).

### BrdU incorporation

A cell proliferation enzyme-linked immunosorbent assay (BrdU kit; Beyotime) was used to analyze the incorporation of BrdU during DNA synthesis following the manufacturer's protocols. Absorbance was measured at 450 nm in the Spectra Max 190 ELISA reader (Molecular Devices, Sunnyvale, CA).

### Flow cytometry analysis

Cells were trypsinized and fixed in 70% ethanol at −20°C overnight. Cells were then resuspended in PBS containing 40 μg/ml propidium iodide and 100 μg/ml RNase A. After incubation for 1 h at 37°C, cells were characterized and cell cycle distribution was determined by fluorescence activated cell sorting (FACS). For each sample, 10000 cells were analyzed. Data was acquired using a BD LSRII apparatus and analyzed using the FlowJo software.

### Colony formation assay

Cells were seeded in a 6-well plate 48 hours posttransduction and cultured for 8 to 10 days at 37°C in 5% CO_2_. Cells were fixed with 4% paraformaldehyde in phosphate-buffered saline (PBS), washed twice with PBS, and stained with a crystal violet solution (1% crystal violet, 10% ethanol in water). Stained cells were washed thrice with water and counted by under an optical microscope.

### Statistical analysis

The data shown represent the mean ± standard error (SE) values of three independent experiments. Significance was analyzed using Student's *t*-test (*p < 0.05, **p < 0.01, ***p < 0.001).

## SUPPLEMENTARY FIGURES


