# Supplementary material for: MicroRNA-153 promotes Wnt/β-catenin activation in hepatocellular carcinoma through suppression of WWOX
Source: Oncotarget. 2015 Feb 2;6(6):3840–7. doi: 10.18632/oncotarget.2927 (PMC4414157; doi:10.18632/oncotarget.2927)
Supplement: Supplementary file 1 [file oncotarget-06-3840-s001.pdf]

SUPPLEMENTARY FIGURES

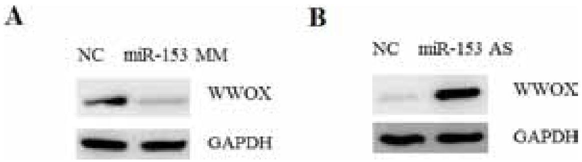

Supplementary Figure 1: (A–B) Western blot analysis of WWOX expression in HuH7 cells transfected with miR-153 mimics (A), antisense (B) or negative control (NC).

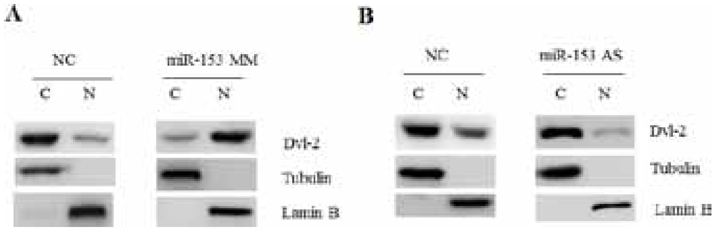

Supplementary Figure 2: (A–B) Cytoplasmic and nuclear protein levels of Dvl-2 in HepG2 cells transfected with miR-153 mimics, antisense or negative control (NC).

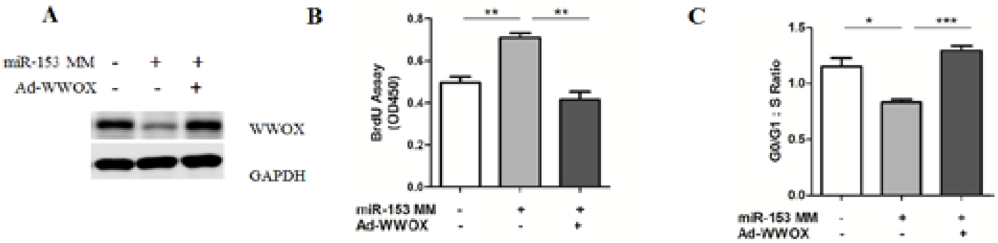

Supplementary Figure 3: (A) WWOX protein expression was determined by Western blot in HepG2 cells. Cells were pre-transfected with miR-153 mimics or negative control (NC) for 24 hr, and then transfected with adenovirus containing WWOX or GFP for another 24 hr. **(B–C)** The cell proliferation (B) and cell-cycle analysis were determined in HepG2 cells as indicated in (A).

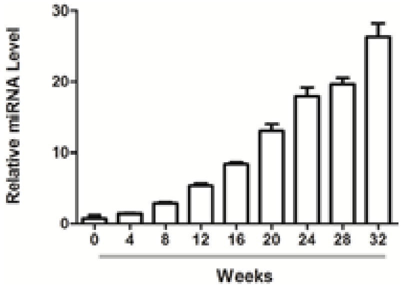

Supplementary Figure 4: Relative expression of miR-153 during DEN-induced liver carcinogenesis in mice.
